# Mitochondrial Calcium: Effects of Its Imbalance in Disease

**DOI:** 10.3390/antiox11050801

**Published:** 2022-04-20

**Authors:** Deyamira Matuz-Mares, Martin González-Andrade, Minerva Georgina Araiza-Villanueva, María Magdalena Vilchis-Landeros, Héctor Vázquez-Meza

**Affiliations:** 1 Departamento de Bioquímica, Facultad de Medicina, Universidad Nacional Autónoma de México, Avenida Universidad 3000, Cd. Universitaria, Coyoacán, Ciudad de México 04510, Mexico; deya@bq.unam.mx (D.M.-M.); martin@bq.unam.mx (M.G.-A.); vilchisl@unam.mx (M.M.V.-L.); 2 Institute of Microbiology, Heinrich Heine University Düsseldorf, 40204 Düsseldorf, Germany; araizavi@uni-duesseldorf.de

**Keywords:** calcium, mitochondria, disease, neurodegenerative

## Abstract

Calcium is used in many cellular processes and is maintained within the cell as free calcium at low concentrations (approximately 100 nM), compared with extracellular (millimolar) concentrations, to avoid adverse effects such as phosphate precipitation. For this reason, cells have adapted buffering strategies by compartmentalizing calcium into mitochondria and the endoplasmic reticulum (ER). In mitochondria, the calcium concentration is in the millimolar range, as it is in the ER. Mitochondria actively contribute to buffering cellular calcium, but if matrix calcium increases beyond physiological demands, it can promote the opening of the mitochondrial permeability transition pore (mPTP) and, consequently, trigger apoptotic or necrotic cell death. The pathophysiological implications of mPTP opening in ischemia-reperfusion, liver, muscle, and lysosomal storage diseases, as well as those affecting the central nervous system, for example, Parkinson’s disease (PD), Alzheimer’s disease (AD), Huntington’s disease (HD), and amyotrophic lateral sclerosis (ALS) have been reported. In this review, we present an updated overview of the main cellular mechanisms of mitochondrial calcium regulation. We specially focus on neurodegenerative diseases related to imbalances in calcium homeostasis and summarize some proposed therapies studied to attenuate these diseases.

## 1. Introduction

Calcium (Ca^2+^) is involved in energy production, cell signaling processes and determines cell fate by triggering or preventing apoptosis. These processes require adequate regulation of this divalent cation’s extra and intracellular concentration since its dysregulation could compromise cell life. Therefore, evolution generated several systems to regulate Ca^2+^, helping the cell to maintain adequate concentrations so that the intracellular metabolic systems perform their function correctly [1]. The concentration of Ca^2+^ in the cytoplasm ([Ca^2+^]_cyt_) is more than 10^4^ times lower than in the extracellular space, through different mechanisms, where ATPases, Ca^2+^ transporters, Na^+^/Ca^2+^ exchangers, and binding proteins are involved. The subcellular levels of Ca^2+^ can vary; in the intracellular organelles known as Ca^2+^ stores, the concentration of this ion is higher than in the cytoplasm (1–5 × 10^−4^ M). It has been described that the main internal store of Ca^2+^ is in the endoplasmic reticulum (ER) and, in muscle cells, the sarcoplasmic reticulum (SR). In the case of mitochondria, Ca^2+^ levels in the nuclear matrix ([Ca^2+^]_n_) and in the mitochondrial matrix ([Ca^2+^]_mt_) are similar to those in the cytoplasm [2].

In eukaryotic cells, mitochondria, cytochrome P450 (CYP450) enzymatic system, peroxisomes, xanthine oxidase (XO), and NADPH oxidases (NOX) are the prominent participants in ROS generation [3,4,5,6,7,8].

However, the highest production of ROS occurs in the mitochondrion, mainly because it has 11 sites that can generate superoxide anion (O_2_^−)^ and hydrogen peroxide (H_2_O_2),_ and six of these sites operate with the NADH/NAD^+^ of the complex I of the respiratory chain. The remaining five operate with the ubiquinol/ubiquinone pair of complex III [9,10]. Mitochondria are organelles present in almost all eukaryotic cells [11]. They are formed by two types of non-contiguous membranes with different functions: outer and inner membranes. The latter has a very sinuous complex structure, forming the mitochondrial cristae. The function of the cristae is to increase the inner membrane surface area where oxidative phosphorylation (OxPhos) occurs [12,13,14,15,16]. Additionally, the inner membrane contains specific systems for the transport of different molecules, such as pyruvate, succinate, malate, amino acids, calcium, and most of the proteins that conform to the complexes that carry out oxidative phosphorylation; only oxygen and carbon dioxide can freely cross this membrane [12,16,17].

Calcium is an essential cation in cells and serves as a second messenger in different signaling cascades regulating cellular functions, including cell growth, contraction, secretion, metabolism, gene expression, cell survival, and cell death [18].

In higher organisms, multicellular calcium homeostasis is regulated in the extracellular fluid, influenced by dietary intake, Ca^2+^ absorption in the small intestine, exchange to and from bone, and Ca^2+^ excretion in the urine [19]. Therefore, the control of Ca^2+^ homeostasis within the cell and between cells is the basis of different physiological processes at the level of the whole organism [20]. On the other hand, at the intracellular level, Ca^2+^ levels are maintained by the sum of the influx of Ca^2+^ that occurs across the plasma membrane (PM) using channels and the release of Ca^2+^ from intracellular stores into the cytoplasm. As mentioned above, the ER and mitochondria are the leading stores of Ca^2+^. The role of mitochondria in calcium homeostasis is essential because of their role as the primary cellular chamber involved in ATP production and because there is a link between mitochondrial calcium levels and mitochondrial dynamics, function, and metabolism. Mitochondria are also involved in cellular microdomains that regulate vital cellular processes [21]. 

The contact sites between the mitochondria and the Ca^2+^ channels present in the ER and the cell membrane create microdomains that allow an increase in the concentration of mitochondrial Ca^2+^ (mCa^2+^). In addition, microdomains between the mitochondria and the cell membrane allow extracellular calcium entry through cell membrane channels into the mitochondria, providing a link between cell activation and mitochondrial function [22].

The interaction between the ER and mitochondria is mediated by docking proteins, and docking plays an important role in multiple cellular pathways. Alterations in these interactions are associated with various diseases, including cancer, metabolic diseases, and neurological disorders [23,24,25].

These physical interactions between the ER and the mitochondria have been determined by electron microscopy; the distance between the outer mitochondrial membrane (OMM) and the ER can vary between 10 and up to 100 nanometers [26]. These microdomains, formed by ER membranes associated with mitochondria (MAM) are highly flexible and can be remodeled with increased cellular and mitochondrial metabolism [27]. An isolated fraction of MAM contains fragments of OMM, the ER, and plasma membrane proteins, clearly reflecting the close link between the ER and mitochondria [28,29,30].

The ER and mitochondria together, through these structures, are involved in the regulation of important physiological processes, such as lipid and Ca^2+^ homeostasis, mitochondrial dynamics, inflammation, and autophagy [31]. Ca^2+^, as a second messenger, plays a major role in normal neuronal activity and brain functions by regulating the synthesis and secretion of neurotransmitters. The intracellular Ca^2+^ concentration is essential for the release of neurotransmitters and is also important for maintaining the postsynaptic action potential [32].

ER ryanodine receptors (RyR) and IP3Rs play an important role in Ca^2+^ homeostasis. Following the action of phospholipase C, IP3 activates IP3Rs, which then release Ca^2+^ from ER stores into the cytoplasm [33,34].

For this reason, the communication between the ER and the mitochondria has received great attention in the field of neurology. The alteration of its anchoring structures is considered a common feature in different neurological disorders. This is due to its extensive involvement in the regulation of many physiological processes.

Under resting conditions, mCa^2+^ concentrations remain low, but after a stimulus, Ca^2+^ is transferred from the SR to mitochondria that transiently and rapidly absorb large amounts of Ca^2+^. Finally, Ca^2+^ is expelled from the mitochondria by the Na^+^/Ca^2+^ antiporter (NCLX), an internal mitochondrial membrane exchanger [35,36].

Other processes that affect calcium homeostasis are the generation of reactive oxygen species (ROS), which can modify the entry and exit of calcium or the homeostasis of other positively charged ions. This, together with the damage that the same ROS can cause within the mitochondria, could cause the cell to enter the process of apoptosis or generate other types of pathologies [37,38]. Here, we review the mechanisms by which mitochondria maintain calcium homeostasis, the relationship between ROS and the dysregulation of calcium levels, and their effects on cell death (apoptosis and necrosis) [39]. Additionally, how the imbalance in the regulatory processes of Ca^2+^ is the main basis of many diseases, and finally, we mention the proposed therapies to treat diseases caused by mCa^2+^ dysregulation.

## 2. Calcium Homeostasis

Cells can detect intracellular changes in calcium concentration (amplitude, duration, frequency, and location) and react accordingly to maintain calcium homeostasis and prevent cell damage. To keep balance, eukaryotic cells have developed mechanisms that can detect changes in calcium concentrations and maintain them by sequestering, buffering, or transporting this divalent ion [20].

However, some mechanisms that carry out calcium homeostasis, such as the buffer system (performed by proteins) and the pumps of some organelles, are only transient and do not affect the long-term calcium concentration. Eukaryotic cells have developed, throughout evolution, mechanisms that temporarily regulate intracellular calcium concentrations. For example, the buffer system made by proteins and Ca^2+^ transporting organelles. Under basal conditions, cytosolic Ca^2+^ levels depend on the balance between Ca^2+^ uptake from the extracellular matrix via PM transporters and Ca^2+^ release (out) or uptake (in) from intracellular stores found in some organelles, such as the mitochondria [21,35].

Changes in Ca^2+^ levels are regulated by internal and external signals, these signals are integrated by an intimate interaction of channels and transporters, and during the last years important components that contribute to this behavior or to the movement of calcium have been identified and characterized. Ca^2+^ signals are translated by an elaborate mechanism dependent on Ca^2+-^ binding proteins, many of which function as Ca^2+^ sensors [40].

Ca^2+^ uptake can occur through the mCa^2+^ uniporter (MCU), while efflux is mediated by NCLX. The uptake and efflux of mCa^2+^ can regulate cytoplasmic Ca^2+^ concentrations both directly and indirectly. Direct regulation occurs by alteration in total Ca^2+^ levels. While indirect regulation occurs because of mitochondrial influence on the activity of SR or plasma membrane Ca^2+^ channels [41,42].

Organelles such as the ER and mitochondria are some of the key players in finetuning control of cytoplasmic calcium levels. Mitochondria are mainly related to energy metabolism and cellular respiration processes, also important in calcium homeostasis and the generation of ROS, which are closely related and have been found to be dysregulated in aging and some neurodegenerative disorders such as Parkinson’s disease (PD), amyotrophic lateral sclerosis (ALS), and frontotemporal dementia disease (FTD) [36,37,38,43]. Calcium homeostasis in mitochondria plays a crucial role in cell physiology and pathophysiology and is critical in cell death [44,45]. Under basal conditions, the mCa^2+^ concentration remains the same as in the cytoplasm (100–200 nM). However, during calcium stimulation, as occurs in neurons and other excitable cells, the mitochondria could accumulate 10–20 times more calcium than the cytoplasm [36].

### 2.1. Buffering of Matrix Calcium

At the intracellular level, Ca^2+^ buffering is a very efficient process, occurring in milliseconds, and any molecule with the ability to bind Ca^2+^ ions could be considered, at least in principle, as a buffer; for example, parvalbumins (α and β isoforms), calbindin-D9k, calbindin-D28k, and calretinin, which have proven Ca^2+^ buffering function and may also have Ca^2+^ sensing functions [46]. All these proteins contain acidic residues in their side chain, which allow them to bind or trap Ca^2+^. However, the mobility of the buffer depends on the molecular weight, concentration, calcium binding affinity or other kinetic constants, as well as the cellular diffusion of said proteins [10]. Ca^2+^ buffers should be viewed as one of the components involved in the precise regulation of Ca^2+^ signaling and Ca^2+^ homeostasis. Each cell is equipped with proteins, including Ca^2+^ channels, transporters, and pumps that, together with Ca^2+^ buffers, shape intracellular Ca^2+^ signals. All these molecules are not only functionally coupled, but their expression is likely regulated in a Ca^2+^-dependent manner to maintain normal Ca^2+^ signaling, even in the absence or malfunction of one of the components [46,47].

### 2.2. Mitochondrial Calcium Uptake

**Figure 1:**
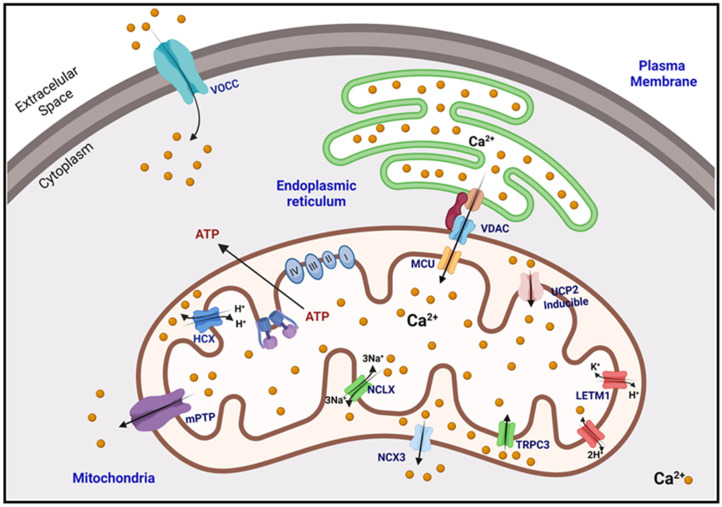
The general model of calcium transport in cells. Anchored in the outer mitochondrial membrane (OMM), are the voltage-dependent transports (VDACs), which mainly uptake calcium from cytosol to the intermembrane space (IMS). However, sometimes exclude calcium as well as the NCX3 transporter and the mitochondrial permeability transition pore (mPTP). In the inner mitochondrial membrane (IMM) calcium is transported to the matrix through some transporters such as the mitochondrial Ca^2+^ uniporter complex (MCU), which is a ubiquitous uniporter. The K^+^/H^+^ or Ca^2+^/H^+^ antiporter leucine zipper containing transmembrane protein 1 (LETM1), the mitochondrial uncoupling protein UPC, and the canonical short transient receptor potential channel 3 (TRPC3). For the exclusion, two primary transporters have been described: the mitochondrial Na^+^/Ca^2+^ ex-changer (NCLX) and the mitochondrial H^+^/Ca^2+^ exchanger (HCX). Mitochondria also present contact sites with the ER and PM, forming zones of high calcium concentration, compared with bulk cytoplasmic calcium, so called microdomains. On the other hand, PM presents some voltage-operated calcium channel (VOCC) transporters nearby contact sites with mitochondria, where the microdomains are formed.

In terms of uptake, it must be considered that cytosolic calcium has to cross two membranes to reach the mitochondrial matrix. The first is the outer mitochondrial membrane (OMM) and then the inner mitochondrial membrane (IMM), both with pores or channel proteins that allow the transport of calcium [36]. The high permeability of the OMM is due in part to the expression and function of VDAC1 (Figure 1), which enables the transport of all energy-related metabolites (i.e., succinate, malate, pyruvate, NADH, ATP, ADP, and phosphate) from the cytosol to the mitochondria [44,45]. There are three subtypes of VDAC (VDAC1, VDAC2, and VDAC3), expressed more or less ubiquitously. However, they vary in the ratio of their isoforms and their concentration in the OMM, depending on the tissue and organ being analyzed [48,49].

These channels can adopt different structural conformations, and as the name implies, the structural transition between the open (2.5 nm) or closed (0.9 nm) pore of VDACs depends on the voltage [50,51]. Several observations support the notion that VDACs can precisely regulate cellular processes in an isoform-dependent manner. Evidence shows that a selective genetic knockout of VDAC subtypes exhibits different phenotypes. Furthermore, VDAC1 and VDAC2 exert opposite effects. While VDAC1 acts predominantly as a proapoptotic protein, VDAC2 protects from various apoptosis inducers [52].

Once in the intermembrane space (IMS), Ca^2+^ ions pass the IMM primarily through the mitochondrial Ca^2+^ uniporter complex (MCU) (Figure 1). The mitochondrial calcium uniporter complex (MCU) is used by mitochondria to take up Ca^2+^ to regulate energy production, cytosolic Ca^2+^ signaling, and cell death. In mammals, MCU is composed of four core components: the pore-forming protein MCU, the gatekeepers MICU1 and MICU2, and an auxiliary subunit, EMRE, essential for Ca^2+^ transport [53], which is composed of subunits that form a pore in the IMM [36]. This uniporter complex is part of a macromolecular complex, which comprises pore-forming proteins and regulatory proteins [54,55,56]. This macromolecular holo complex is composed of the MCU as mentioned above (previously known as CCDC109a) as a channel-forming component. The regulator MCUb (also known as CCDC109b) [57] and the mitochondrial calcium uptake protein 1 (MICU1) [58] which binds to its paralog, MICU2, to form a heterodimeric structure. MICU1-MICU2 dimers act as uniporter gatekeepers, setting the Ca^2+^ concentration threshold for MCU activation and allowing mitochondrial Ca^2+^ uptake exclusively at high calcium concentration; thus limiting the deleterious accumulation of mitochondrial matrix calcium in basal conditions [59,60,61]. This heterodimer is associated with MCU through the essential regulator of MCU (EMRE) (also known as SMDT1) [62].

The MCU complex is unequivocally the dominant mechanism that enables calcium uptake into the mitochondrial matrix [36]. However, MCU-independent mechanisms of Ca^2+^ uptake have been described, including the canonical short transient receptor potential channel 3 (TRPC3) [63], the mitochondrial uncoupling proteins 2 and 3 (UCP 2–3) [64], and leucine zipper containing transmembrane protein 1 (LETM1) [65]. LETM1 is a protein found in the IMM, which is required to maintain mitochondrial morphology and cristae structure (Figure 1) [66]. It is also a mitochondrial K^+^/H^+^ and mitochondrial Ca^2+^/H^+^ antiporter that contributes to Ca^2+^ influx when extramitochondrial Ca^2+^ concentrations are relatively low (<1 μM) [64]. The LETM1 family protein plays a central role in regulating mitochondrial cation transport and osmotic volume. This family has been found in all eukaryotes, and a highly conserved protein architecture has been demonstrated in different organisms. LETM1 has been identified as an antiporter that exchanges Ca^2+^ or K^+^ for H^+^. This last point has been controversial since it is still unknown if this transporter is mainly a K^+^/H^+^ exchanger (KHE), as initially proposed or a Ca^2+^/H^+^ exchanger (CHE) as subsequently demonstrated [67,68].

Transient receptor (TRP) channels have been described as important cellular sensors. Although many TRP channels are expressed at the plasma membrane, some members of the TRP channel proteins are also found in intracellular organelles, such as the ER, secretory vesicles, granules, endosomes, lysosomes, and mitochondria [69,70,71,72,73]. Studies have shown that TRPC3 interacts with many mitochondrial proteins [74], confirming that the TRPC3 protein is located in this organelle [63]. Although MCU and LETM1 affect mitochondrial Ca^2+^ uptake in a Ca^2+^ concentration-dependent manner, TRPC3 plays an important role in mitochondrial Ca^2+^ uptake (Figure 1). This was confirmed by a study in which overexpression of TRPC3 in HeLa cells led to a substantial reduction in cytosolic Ca^2+^. In contrast, cytosolic Ca^2+^ increased in cells expressing the mutant TRPC3 (E630Q) [75]. It has been described that TRPC3 favors Ca^2+^ entry into the matrix when the concentration of this divalent cation is high (≥50 μM) [63].

Other necessary transporters are UCPs, described to transport protons against the electrochemical gradient through the IMM; thus decoupling the proton gradient from ATP production. Although it may seem counterproductive, this uncoupling process has several physiological functions. For example, uncoupling protein 1 (UCP1) uncouples the proton gradient in brown adipose tissue to generate heat. Other functions of uncouplers are to reduce the formation of ROS and maintain the NAD^+^/NADH ratio [76]. The UCP transporter family is composed of five members (UCP 1–5) in mammals, and these are differentially expressed in tissues and organs [77]. UCP1 is mainly expressed in brown adipose tissue.

In contrast, UCP2 is ubiquitously expressed and abundant in the liver, kidney, pancreas, spleen, neurons, and skeletal muscle. At the same time, UCP3 is predominantly found in skeletal muscle and cardiac cells, while UCP4 and UCP5 are more frequent in neurons [78]. In the case of UCP2 and UCP3, it was shown by overexpression, knock-down, and mutagenesis that the overexpression of UCP2 or UCP3 significantly increased Ca^2+^ sequestration by mitochondria (Figure 1), while its deletion drastically reduced it [64].

It has been suggested that UCP2 might somehow regulate the activity of other transporter proteins. This was confirmed by showing that UCP2 could alter the stability of the mitochondrial cristae junction, which is maintained by oligomerized MICU1; leading to proton leakage out of the cristae lumen into the IMS, explaining the uncoupling function of UCP2, in addition to cristae leakage leading to easier access of calcium to the cristae; thus, increasing mCa^2+^ in the matrix [78,79]. This review mentions some of the leading transporters responsible for mitochondrial calcium uptake. However, there are many more diverse proteins related to this process, such as the mitochondrial ryanodine receptor (mRyR) and rapid absorption of mitochondrial calcium (RaM) [80]. The ryanodine receptor (RyR) is a channel that releases Ca^2+^ from the sarcoplasmic reticulum (RS) to the cytoplasm, these ions then bind to troponin C, which is on the microfilaments; thus initiating muscle contraction. This process of calcium release is known as Ca^2+^-induced Ca^2+^ release (CICR) [81]. The RyRs belong to a highly conserved family of Ca^2+^ release channels. Three RyR isoforms encoded by three different genes have been described (Ryrl, Ryr2, Ryr3); the RyRl isoform found in fast-twitch skeletal muscle, the RyR2 isoform expressed in heart muscle and brain, and the RyR3 isoform expressed in smooth muscle and brain. The RyRs form the Ca^2+^ release channel by generating a tetrameric structure; in this conformation it is the largest ion channel described so far, with a weight of ~2000 kDa. Approximately 90% of each of the channel subunits form an amino-terminal region, which is located towards the cytosol and has regulatory functions [81,82].

### 2.3. Mitochondrial Calcium Extrusion

Two main systems have been described to transport Ca^2+^ from the mitochondrial matrix to the IMS: the mitochondrial Na^+^/Ca^2+^ exchanger (mNCX) and the mitochondrial H^+^/Ca^2+^ exchanger (HCX). Mitochondrial NCX was initially described as only a Na^+^/Ca^2+^ antiporter. However, it was found to transport lithium (Li^+^) or sodium (Na^+^) in exchange for Ca^2+^, while PM exchangers do not transport Li^+^. For this reason, it was later called NCLX as a shorthand term for (Na/Li/Ca exchanger) [83]. NCLX is the predominant antiporter in excitable tissues in the heart and brain, while HCX is found in non-excitable tissues such as the liver and kidney. The stoichiometry of the NCLX transporter is three (or four) Na^+^/Li^+^ for each Ca^2+^ [84,85], while HCX exchanges two H^+^ for one Ca^2+^ (Figure 1) [86]. Calcium extruded by the systems mentioned in the IMS is then transported to the cytoplasm via VDAC or other Ca^2+^ extrusion mechanisms also located in OMM, which is NCX3 [87]. It has also been proposed that, under certain conditions, another alternative for calcium efflux is the transient opening of a mitochondrial permeability transition pore (mPTP). Opening of the mPTP means that the inner mitochondrial membrane no longer represents a barrier to protons, leading to dissipation of the force provided by the proton gradient. The resulting uncoupling of oxidative phosphorylation then leads to a cessation of ATP production by the mitochondria, with the consequent energy deficit for cellular functions. Three proteins have been proposed as structural components of the mPTP: the potential-sensitive anion channel (VDAC) located in the outer mitochondrial membrane, the adenine nucleotide transporter (ANT) in the inner membrane, and Cyclophilin D (CypD) in the matrix mitochondria [36].

### 2.4. Microdomains, Connections of the Mitochondria with the ER and the PM

As mentioned above, the leading calcium transporter in the mitochondrial matrix is MCU. However, it is known to have a low affinity for calcium. It requires high concentrations of this ion for its transport, so the microdomain hypothesis explains how the cytoplasmic transport of calcium could be carried out at low calcium concentrations [88].

This hypothesis states that rapid mCa^2+^ uptake in intact cells depends on the proximity between the mitochondria and the calcium release/uptake sites of the ER or extracellular calcium influx at the PM. These sites may experience local changes. Calcium concentrations are much higher than those measured in the cytosol, sufficient to trigger the calcium uptake mechanism in the mitochondria [89]. There is evidence of this proximity; even contact sites between mitochondria with ER or PM have been described using high-resolution microscopy techniques and electron microscopy [90,91]. It should be noted that the mitochondrial populations within a cell can be divided into two populations; one located in close contact with calcium release sites and others not located at these sites; thus experiencing lower calcium concentrations closer to those found in the cytosol [92].

Calcium uptake/efflux into these microdomains is mediated by ER/PM transport proteins that couple calcium transport to mitochondria. Using epifluorescence microscopy and TIRF microdomains of PM-mitochondria were found to occur once PM voltage-operated calcium channels (VOCCs) were reported to open. This VOCC transporter carries calcium from the extracellular space to the cytosol, which is then taken up by the mitochondria (Figure 1) [93]. The contact sites of the ER and the mitochondria locally transmit calcium signals between the IP3 receptors (IP3R) and the mitochondrial calcium uniporter and are essential for cell survival [94]. IP3Rs are high-conductance cation channels that mediate Ca^2+^ release from the ER, giving rise to cytoplasmic calcium that is effectively transported into the mitochondrial matrix [95,96].

There are other anchoring proteins that are essential components for the formation of microdomes related to calcium homeostasis between the ER, the mitochondria, and the plasma membrane. It has been reported that some modifications or alterations of these proteins are closely linked to the development of different neurodegenerative diseases. Here are some examples:

#### 2.4.1. Presenilins

Presenilin 1 and 2 (PS1 and PS2) are proteins found constitutively and enriched in MAM [30]. PS2 has been reported to enhance the number of contact sites between the ER and the mitochondria; these are accompanied by mitofusin 2 (MFN2), which functions as a key link between the ER and the mitochondria [97]. Furthermore, PS are key components in the formation of the γ-secretase complex, involved in the production of amyloid β (Aβ) peptides, which are the main pathological hallmark of AD [98].

Moreover, PS have been shown to play a vital role in Ca^2+^ homeostasis within the ER, as some FAD-linked PS2 mutants have altered expression of ER Ca^2+^ release channels, as well as IP3R and RyR. PS without alterations or mutations (WT) were also found to form low-conductance Ca^2+^ leak channels in the ER membrane [99]. Therefore, since PS2 promotes Ca^2+^ transfer between the ER and mitochondria, an overexpression of PS2 mutants promotes an increase in Ca^2+^ transfer, generating an imbalance in Ca^2+^ homeostasis [100]. Finally, as AMS present a high concentration of PS, it has been concluded that they are related to most neurodegenerative diseases, especially in AD [101].

#### 2.4.2. IP3R–SIG1R–GRP75–VDAC

The IP3R found in the ER membrane and the VDAC present in the OMM, and which are near each other, is mediated by the chaperone GRP75 [102]. With the help of this proximity, Ca^2+^ is transferred from the ER to the OMM and subsequently to the IMM via the MCU [103]. On the other hand, the sigma-1 receptor (SIG1R) is a Ca^2+^-sensitive ER protein, which forms a complex with GRP75 and prolongs Ca^2+^ transfer to mitochondria from the ER by stabilization with IP3R [102]. There are three isoforms of IP3R, of which type 3 isoform is enriched in MMA compared to type 1 and type 2 isoforms [104]. Any mutation/alteration in these proteins leads to changes in the junction between the ER and the mitochondria, which leads to a deregulation of Ca^2+^ mobilization. This has been shown in different studies; for example, overexpression of cytosolic GRP75 increases inositol triphosphate (IP3)-induced Ca^2+^ concentration in mitochondria by stabilizing the binding between IP3R1 and VDAC [102]. Furthermore, overexpression of VDAC1 in HeLa cells enhances the contact efficiency between the ER and the mitochondria and thus increases the concentration of Ca^2+^ in the mitochondria [105]. Another example is that overexpression of SIG1R inhibits the ER stress response and the progression of apoptosis [106]. These proteins are very important in the regulation of Ca^2+^ signaling between the ER and the mitochondria, allowing the bioenergetic response, survival, or cell death [107].

#### 2.4.3. Other Crosstalk Proteins

In addition to the anchoring proteins, there are other proteins that play a crucial role in the junction between the mitochondria and the ER, including vesicle-associated membrane protein-associated protein B (VAPB), a protein found in the ER and protein tyrosine phosphatase-interacting protein-51 (PTPIP51), a protein found in the OMM [108]. Genetic alterations in these proteins are linked to changes in Ca^2+^ homeostasis between the ER-mitochondria and its mobilization to modulate or activate different metabolic processes. Mutations or alterations between these two anchor proteins (VAPB-PTPIP51) have been reported in ALS [109]. In mammals, PTPIP51 interacts with oxysterol-binding protein-related proteins 5 and 8 (ORP5 and ORP8), which regulate cortical contacts between the ER and the plasma membrane [110].

PDZ domain-containing protein 8 (PDZD8) is another anchor protein, crucial for Ca^2+^ homeostasis between the ER and mitochondria, especially for ER-dependent mitochondrial Ca^2+^ uptake. It has been reported that a decrease in the expression of PDZD8 produces the separation of the mitochondria from the ER, which, in turn, causes an increase in the cytosolic levels of Ca^2+^. This demonstrates that PDZD8 is crucial for synaptic signaling [111].

## 3. Oxidative Stress Generated by Changes in Mitochondrial Calcium Concentration

Oxygen is indispensable for life. However, its intermediates can be disease-generating sources by uncontrolled production of ROS and free radicals (FRs) that can damage the structure of organelles and macromolecules such as lipids, proteins, carbohydrates, and nucleic acids [112,113]. When the reduction of molecular oxygen (O_2_) is partial superoxide anion (O_2_^−^) radical is formed and it has been estimated that 0.2–2.0% of the O_2_ consumed by mitochondria is converted to O_2_^−^ [3,114,115]. Thus, when O_2_ captures an electron, O_2_^−^ is produced. Eventually, O_2_^−^ can produce H_2_O_2_ and the hydroxyl radical (OH^−^) [116,117]. An excess of FRs breaks the cell’s oxide-reduction equilibrium, producing the so-called oxidative stress. This oxidative stress is a cellular phenomenon or condition that occurs because of a physiological imbalance between the levels of antioxidants and oxidants (free radicals or reactive species) to favor oxidants [118,119]. Moreover, it is also associated with aging-related diseases [120]. 

The Ca^2+^ ions enter the cell through transmembrane proteins or the so-called calcium channels, either voltage-dependent or receptor-operated. Furthermore, specialized calcium transport systems are located in cellular organelles such as mitochondria, nucleus, and Golgi apparatus [121]. For example, mCa^2+^ uptake is electrogenic, driven by the voltage present in the inner mitochondrial membrane product of the proton pumping performed by the respiratory chain. This transport can be accomplished in different ways, either through the mPTP, MCU, or NCLX [121]. In mitochondria, Ca^2+^ homeostasis plays a crucial role in physiology, cellular pathophysiology, and is critical for cell death [37,38].

Additionally, Ca^2+^ in mitochondria promotes ATP synthesis, which results from stimulation of Krebs cycle enzymes and OxPhos [122]. This effect is achieved by physiological Ca^2+^ signals and allows adjustment of ATP production to cellular demand. However, Ca^2+^ also has adverse effects on mitochondria since elevated Ca^2+^ levels activate ROS-generating enzymes and FRs formation [121,123,124]. Therefore, the interactions between ROS and calcium signaling can be considered bidirectional, as ROS can regulate cellular calcium signaling, while calcium signaling is essential for ROS production [125]. However, the interaction and mutual communication of reactive oxygen species and calcium depend on cell types and tissues to a large extent. So far, this interaction has been studied mainly in the cardiovascular system and brain, where ROS and Ca^2+^ signals act on the same cellular targets [122,126].

As already mentioned, mCa^2+^ mainly promotes ATP synthesis by stimulating the activity of the enzyme pyruvate dehyrogenase and the limiting dehydrogenases of the Krebs cycle (isocitrate dehydrogenase, α-ketoglutarate dehydrogenase); as well as OxPhos, and consequently increased oxygen consumption, which would lead to increased electron leakage from the respiratory chain and increased generation of ROS by complexes I and III [17,123]. Indeed, mitochondrial ROS generation was correlated with metabolic rate [127]. There is evidence to indicate that the metabolic state of mitochondria determines the effects of calcium on the levels of ROS they generate.

More specifically, when mitochondria are at rest (state IV), they are characterized by low electron flow and ATP synthesis, low rates of O_2_ consumption, and a high NADH/NAD^+^ ratio leading to high ROS production. On the other hand, when mitochondria are synthesizing ATP (State III), the opposite occurs, high electron flow and ATP synthesis, high O_2_ consumption rates, and low NADH/NAD^+^ ratio, which results in lower ROS production [3,128,129,130]. However, it should be noted that mCa^2+^ overload increases ROS production independently of the metabolic state of mitochondria [3,115,131]. In this regard, it is known that a mCa^2+^ overload triggers several processes, including the opening of mPTP and this, in turn, leads to the collapse of the mitochondrial membrane potential and a transient increase in ROS generation [121,132], which gives rise to a process called ROS-induced ROS release (RIRR). The release of ROS in the cytosol can lead to activation of RIRR in neighboring mitochondria due to ROS trafficking between mitochondria. It is likely that from the generated ROS H_2_O_2_ is the messenger molecule leading to RIRR, due to its longer lifetime in the cytosol and its higher permeability in membrane lipids which could constitute a positive feedback mechanism for increased ROS production that could lead to significant mitochondrial and cellular injury [121,133,134]. RIRR was initially described in animal cells and proposed to mediate mitochondrion to mitochondrion communication but was later expanded to include communication between mitochondria and plasma membrane-localized NOX. It should be noted that Ca^2+^ also stimulates the activity of this enzyme and since the biological function of these enzymes is to produce ROS for signaling purposes, specifically O_2_, this would partly explain the increased production of reactive oxygen species in mitochondria [135,136]. In summary, stress conditions leading to calcium or ROS overload trigger mPTP opening, leading to loss of mitochondrial membrane potential; this fails in ATP production and release of mitochondrial proteins such as cytochrome c, which trigger cell death through necrotic and apoptotic pathways, respectively [17,132].

## 4. Alterations of Mitochondrial Ca^2+^ (mCa^2+^) Signaling in Neurodegenerative Disorders

**Figure 2:**
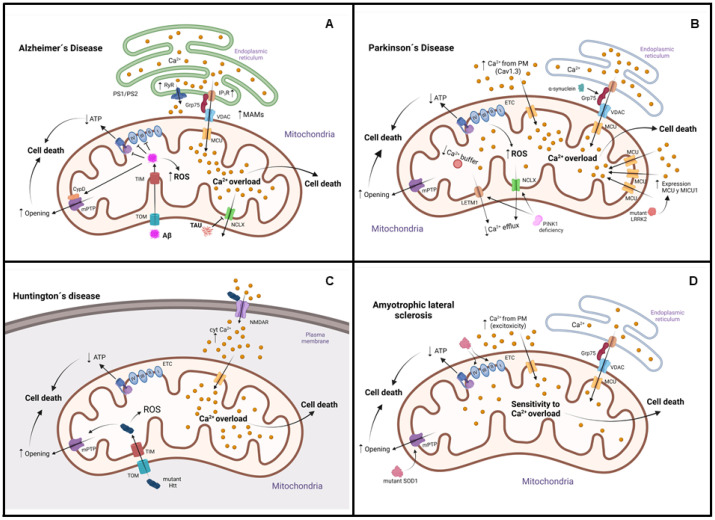
Dysregulation of mCa^2+^ in neurodegenerative disorders. (**A**) In Alzheimer’s disease, amyloid-beta aggregates (Aβ) enter mitochondria through the translocases TOM and TIM. Inside, they interact with respiratory chain complexes III, IV, and ATP synthase, leading to ATP synthesis reduction and with CypD, leading to mPTP opening, potential membrane collapse, and cell death activation. Additionally, there is an excessive level of mCa^2+^ caused by increased IP3Rs and RyR activity dysregulation of voltage-operated channels. Moreover, Tau protein decreases mCa^2+^ efflux by suppressing NCLX. (**B**) In Parkinson’s disease, α-synuclein interacts with the chaperone glucose-regulated protein 75 (Grp75), increasing endoplasmic reticulum–mitochondria communication and mCa^2+^. PTEN-induced kinase 1 (PINK1) deficiency results in mitochondrial calcium overload and ROS production by decreased activity of NCLX and LETM1. (**C**) In Huntington’s disease, mHtt (mutant huntingtin) enters mitochondria through the translocases TOM and TIM and causes NMDA receptor overactivation in PM, resulting in increased calcium influx and significant mitochondrial depolarization, and ATP synthesis reduction. Additionally, mHtt decreases the Ca^2+^ threshold necessary to trigger mPTP opening. (**D**) In amyotrophic lateral sclerosis, SOD1 mutant has an early increase in mCa^2+^ by endoplasmic reticulum overload and increased cytosolic calcium, which causes loss of mitochondrial membrane potential. In addition, this mutant decreases the activity of respiratory chain complexes I, II, and IV and reduces ATP synthesis. There is an increased mitochondrial susceptibility to Ca^2+^ overload.

mCa^2+^ is crucial in mitochondrial function. An excess of mCa^2+^ can disrupt mitochondrial respiration, increase the generation of ROS, and result in cell death. In neurodegenerative diseases (NDDs) such as Alzheimer’s disease [137], Parkinson’s disease [138], and Huntington’s disease [139], many alterations in mCa^2+^ handling have been observed. Furthermore, mitochondrial changes that induce energy deficit in the brain are present in the asymptomatic stage of the disease before clinical symptoms appear. Thus, mitochondrial metabolic defects could initiate the neurodegenerative process (Figure 2).

### 4.1. Alzheimer’s Disease (AD)

AD is a progressive condition that causes dementia. It is characterized by irreversible memory loss, dysconnectivity, and cell death. Changes in essential metabolic enzymes may impair the neuron’s ability to create ATP via OxPhos, resulting in cellular stress in this disease. It can be early-onset, known as familial AD (FAD) with a prevalence of 10% and mutations in genes such as amyloid precursor protein (APP), presenilin-1 (PS1), and presenilin-2 (PS2). It is an autosomal dominant disease. The other form is late-onset or sporadic (SAD), with a prevalence of 90%. SAD develops after 60 years of age, and it is characterized by alterations in some genes, such as the apolipoprotein E gene (APOE), the most studied [140]. Amyloid-beta (Aβ) and hyper-phosphorylated tau accumulation are histopathological characteristics of the disease. Aβ mutations cause deposition due to overproduction, incorrect cleavage, and accumulation. Aβ aggregation can trigger many events, including oxidative stress, inflammation, neuronal calcium dysregulation, metabolic alterations, and neuronal cell death [141]. The Aβ can enter mitochondria via the translocases of the outer membrane (TOM) [142], and the inner membrane (TIM) interacts with proteins and impairs the activity of respiratory chain complexes III and IV and ATP synthase [143]. Furthermore, this peptide causes mCa^2+^ overload in cell models by increased inositol 1,4,5-trisphosphate receptor activity and dysregulation of voltage-operated channels [144]. Recent studies have shown that cyclophilin D (CypD), a well-known mPTP component, interacts with Aβ and promotes mPTP opening and potential membrane collapse, leading to mitochondrial and neuronal stress and thus causing cell death [145].

In addition, tau protein plays an important physiological role in assembling and stabilizing microtubules. Tau suppresses NCLX function, decreasing mCa^2+^ efflux causing apoptotic cell death [146]. Various models of AD show that dysregulation in mitochondrial calcium may be due to increased ER–mitochondrial connectivity. This causes an overload of calcium in the mitochondria, excess ROS, the opening of the mPTP, and cell death. On the other hand, PS1 and PS2 mutations have been associated with FAD. It has been shown that calcium transfer between the ER and mitochondria is altered by these mutations [137]. PS2 and APP mutations have increased the amount of mitochondria-associated ER membranes (MAMs) [137,147]. They increase expression or sensibility of two main ER calcium release channels, inositol 1,4,5-trisphosphate receptors (IP3Rs) and ryanodine receptors [148,149]. Additionally, Presenilin deletion improves ER-to-mitochondria calcium uptake in Caenorhabditis worms, which do not synthesize Aβ peptides, implying that this protein probably affects ER–mitochondria calcium transfer via an Aβ-independent mechanism [150]. Finally, in the brain of AD patients, there is a decline in enzyme activity of mitochondrial metabolism. As a result, a deficiency of acetyl-CoA reduces the neurotransmitter acetylcholine (ACh) generation. Decreased ACh synthesis induces cholinergic neurotransmission deficits in AD [151,152]. These findings show an additional relationship between energy depletion and neural dysfunction in this disease. In addition, the mCa^2+^ concentration regulates some mitochondrial dehydrogenases such as pyruvate dehydrogenase, α-ketoglutarate dehydrogenase, and isocitrate dehydrogenase; thus participating in mitochondrial metabolism [153,154]. The data obtained suggest that the central mediator of the progress of this AD is the neuronal overload of mCa^2+^. This increased mCa^2+^ impaired mitochondrial metabolism, ATP production, mitochondrial transport, and mPTP opening. All these alterations cause loss of synaptic function and cell death (Figure 2A).

### 4.2. Parkinson’s Disease (PD)

PD is the second most prevalent NDD it is affecting 1–3% of the population above 60 years of age. It is characterized by involuntary shaking, slowness of movements, rigidity, depression, anxiety, fatigue, and dementia. It is caused by a diminishment of the neurotransmitter dopamine and the deposition intraneuronal of Lewy bodies mainly composed of α-synuclein [138]. Among neurodegenerative disorders, PD is probably the most linked to mitochondrial dysfunction. Dopaminergic neurons require 20 times more energy than other neurons, making them more sensitive to mitochondrial malfunction and cell death [155]. Defects in mitochondrial respiration, pyruvate oxidation, complex I, II, and III activity, and mitochondrial transcription factor A (TFAM) have been found in PD patients [156]. Reduced electron transport chain capacity in PD may cause a decrease in ATP and induce vesicular dopamine accumulation [157]. A decrease in ATP may also affect axonal transport [158] and mitochondrial dynamics such as fusion, fission, biogenesis, transport, and ATP-dependent protein degradation [159]. On the other hand, different PD cell models have shown increased mCa^2+^ absorption and reduced export, resulting in mCa^2+^ overload. 

In some cases, mCa^2+^ overload has been attributed to sustained cytosolic Ca^2+^ levels [160]. Indeed, the opening of PM L-type Cav1.3 Ca^2+^ channels causes autonomous, rhythmic peacemaking in dopaminergic neurons [161]. Aging and probably other stresses connected to PD, such as genetic abnormalities, augment this activity [162]. Furthermore, these neurons have a limited Ca^2+^ buffering system, and the expression of calbindin (Ca^2+^-buffering protein) in patients decreases mesencephalon degeneration [163]. All these factors cause an increase in mCa^2+^ uptake and OxPhos activation. Although this could be a process that ensures practical ATP synthesis, when ATP levels are high enough, it can lead to mitochondrial hyperpolarization, ROS formation, mitochondrial DNA damage, and the development of defective organelles [161]. On the other hand, mutations in some genes such as parkin (PRKN) [164] and phosphatase and tensin homolog (PTEN)-induced kinase 1 (PINK1) [165] cause familial PD These proteins help to regulate mitochondrial function. For example, reduced NCLX activity has been associated with elevated mCa^2+^ in many PD models. PKA phosphorylates NCLX in Ser258 and activates it, but it requires PINK1 for its complete activation. As the NCLX-dependent mCa^2+^ efflux is reduced in neurons with PINK1 deficiency, this causes mCa^2+^ overload, mPTP opening, mitochondrial oxidative stress, lower OxPhos, and cell death [166,167]. Furthermore, it has been postulated that PINK1 phosphorylates and activates LETM1. The production of phospho-LETM1 mutants in PINK1-KO neurons lowers mitochondrial Ca^2+^ excess and protects against cell death [168]. Similarly, a lack of LRRK2, another gene linked to familial PD, lowers mCa^2+^ extrusion, a characteristic that can be reversed by increasing NCLX expression [169]. Additionally, mutant LRRK2 induces transcriptional upregulation of MCU and mitochondrial calcium uptake (MICU1); thus leading to mCa^2+^ accumulation [170]. Another protein implicated in PD pathogenesis is the α-synuclein, whose accumulation in Lewy bodies is a hallmark feature of PD. Although its specific roles are unknown, α-synuclein has been linked consistently to pre-synaptic processes and intracellular vesicle movement [171]. This protein interacts with the chaperone Grp75; thus contributing to ER–mitochondria communication [172]. These data suggest that mCa^2+^ excess is a critical stage in the etiology of PD and that manipulating the uptake and efflux machinery could provide promising therapy options (Figure 2B).

### 4.3. Huntington’s Disease (HD)

HD is a neurodegenerative process that causes mental, behavioral, motor, and cognitive problems. HD is characterized by CAG triplet expansion in the htt gene, which codes for huntingtin (Htt) [139]. Several investigations have indicated that mitochondrial dysfunction plays a crucial role in the pathogenesis of HD, even though the fundamental pathways for this illness beginning remain unknown. This disease has been linked to mitochondrial abnormalities ranging from altered membrane potential to structural alterations and defective trafficking [173]. The CAG repeat in the huntingtin gene has been demonstrated to cause NMDA receptor overactivation, which results in increased calcium influx, significant mitochondrial depolarization, ATP synthesis reduction, and excitotoxicity [174]. These conditions are also linked to NOX activation and ROS production [175].

On the other hand, mCa^2+^ has been reported to increase or inhibit uptake, depending on the experimental model. Both lymphoblast mitochondria from HD patients and brain mitochondria from transgenic mice expressing mutant Htt (mHtt) have a decreased mitochondrial membrane potential, with depolarization at lower Ca^2+^ loading [176]. However, isolated brain mitochondria from the transgenic YAC128 mouse model have greater Ca^2+^ uptake capacity and resistance to Ca^2+^-mediated damage [177]. Additionally, similar mitochondrial Ca^2+^ dynamics are observed in isolated brain mitochondria and cultured primary striatal neurons from WT and R6/2 mice expressing a mutated N-terminal fragment of Htt [178]. These conflicting findings may depend on the experimental procedures adopted, and the mechanisms that lead to possible alterations in the handling of mCa^2+^ in HD have not been fully elucidated (Figure 2C).

### 4.4. Amyotrophic Lateral Sclerosis (ALS)

ALS is a neurodegenerative disease that predominantly degrades motor neurons and skeletal muscle dystrophy [179]. Several genes have been linked to the familial forms of the disease (fALS), with the mutations in chromosome-9-open-reading-frame-72 (C9orf72), superoxide dismutase 1 (SOD1), transactive response DNA-binding protein 43 (TDP43), fused in sarcoma (FUS), and Sigma-1R being the most frequent [180]. Some evidence obtained from different ALS models demonstrates decreased ER–mitochondria physical/functional coupling, with a reduced Ca^2+^ transfer and thus a lower mitochondrial ATP synthesis [181]. However, it has been described that there is a greater vulnerability to mCa^2+^ overload in the brain and spinal cord of fALS-SOD1 mutant mice [182]. This effect may be related to glutamate-induced Ca^2+^ excitotoxicity in ALS patients [183]. The spinal cord mitochondria of these mutant mice are more susceptible to oxidative stress and Ca^2+^-induced mPTP opening, causing apoptotic or necrotic death of neurons [184]. Additionally, a more outstanding production of cytosolic Ca^2+^ microdomains, mediated by mPTP-opening, is found in motor cortex astrocytes of symptomatic SOD1 mutant mice [185]. This mutant also can clump together in mitochondria, bind to Bcl-2, lowering its anti-apoptotic effects [186], and decrease the activity of respiratory chain complexes I, II, and IV [187]. Furthermore, motor neurons expressing mutant SOD1 have an early increase in mCa^2+^, which is accompanied by a loss of mitochondrial membrane potential, mitochondrial enlargement, ER overload, and an increase in cytosolic calcium [188]. Therefore, mitochondrial functions are affected by the mutant SOD1 protein. Thus, in ALS, a complex cascade between defective ER–mitochondrial Ca^2+^ communication and increased mitochondrial susceptibility to Ca^2+^ overload may be present at disease onset and progression (Figure 2D).

### 4.5. Other Neurodegenerative Diseases

In spinocerebellar ataxia associated with a lack of the AFGeL2 subunit of the mitochondrial m-AAA protease has been observed and accumulation of mitochondrial calcium uniporter–essential MCU regulator (MCU-EMRE) complexes. These complexes can promote excess mCa^2+^ and mPTP opening, leading to Purkinje cell death in this disease [189]. Patient-derived fibroblasts show higher basal Ca^2+^ levels, disturbing Ca^2+^-modulated processes, such as synaptic transmission and muscle contraction [190]. This disease emphasizes the importance of mCa^2+^ homeostasis for neuronal health. 

Another neurodegenerative illness is Friedreich’s ataxia. It is caused by a decrease in frataxin which is a mitochondrial protein. Frataxin’s function is unknown; however, it appears to be involved in forming iron–sulfur clusters. A frataxin-deficient neuronal model demonstrates changes in calcium-mediated signaling pathways and a decrease in NCLX and apoptosis. Frataxin overexpression in adipocytes increases mCa^2+^ uptake by MCU, tricarboxylic acid cycle upregulation, higher mitochondrial membrane potential, and ATP generation [191]. Additionally, the frataxin-deficient neuronal model obtained from rat dorsal root ganglia showed calcium dysregulation, caspase 3 activation, and apoptotic cell death by increasing mitochondrial calcium buffer capacity [192].

## 5. Therapeutic Approaches

**Figure 3:**
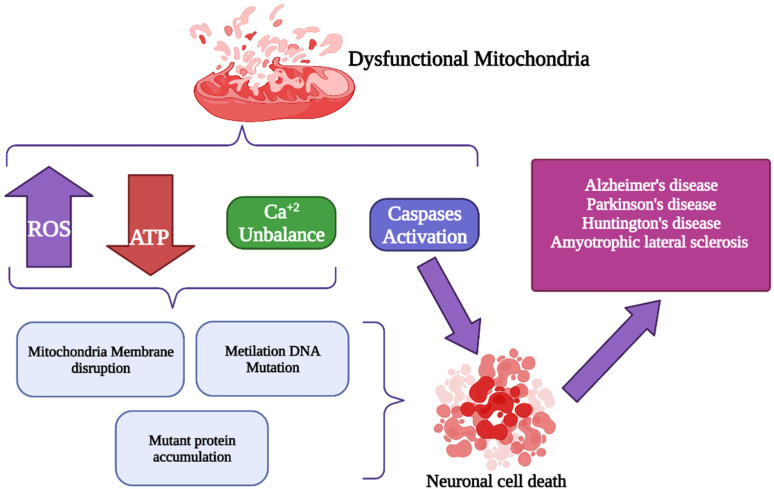
Mitochondrial dysfunction in neurodegenerative diseases. Neurodegenerative diseases such as Alzheimer’s disease, Parkinson’s disease, Huntington’s disease, amyotrophic lateral sclerosis, among others, involve altered signaling of apoptotic proteins, ROS, ATP, and Ca^+2^ imbalance mechanisms. This pathological imbalance is responsible for mitochondrial dysfunction in neurological disorders.

There are different therapeutic approaches, from palliative therapy, guided by good medical practices, including some anticonvulsant drugs, control of endocrine dysfunction, and surgical procedures. Removal of noxious metabolites is centered on combating lactic acidosis but extends to other metabolites. Attempts to bypass blocks in the respiratory chain by administration of electron acceptors have not been successful, but this may be amenable to genetic engineering. The administration of metabolites and cofactors is the mainstay of real-life therapy. It is imperative in disorders due to primary deficiencies of specific compounds, such as carnitine or coenzyme Q10 [193]. There is increasing interest in administering reactive oxygen species scavengers both in primary mitochondrial diseases and neurodegenerative diseases directly or indirectly related to mitochondrial dysfunction. Figure 3 represents the mitochondrial dysfunction resulting from a low level of ATP, resulting in a decrease in the electron transport chain enzymes, generation of ROS, reduction in mitochondrial DNA (mtDNA), and the release of caspase 3. Aerobic exercise and physical therapy prevent or correct deconditioning and improve exercise tolerance in patients with mitochondrial myopathies due to mtDNA mutations [194,195]. Gene therapy is a challenge because of polyplasmy and heteroplasmy. However, interesting experimental approaches are being pursued and include, for example, decreasing the ratio of mutant to wild-type mitochondrial genomes (gene shifting), converting mutated mtDNA genes into normal nuclear DNA genes (allotopic expression), importing cognate genes from other species, or correcting mtDNA mutations with specific restriction endonucleases. Germline therapy raises ethical problems but is being considered to prevent maternal transmission of mtDNA mutations [195,196]. Finally, Table 1 shows some drugs used in mitochondrial dysfunction.

The drugs used in mitochondrial diseases are specific depending on the pathology caused by mitochondrial dysfunction. In AD, oxidative stress, cell death and mitochondrial dysfunction are the main causes of the pathology. In this case, drugs that have been used satisfactorily are curcumin, ferulic acid, ginkgolides, bilobalides, epigallocatechin-3-gallate, ginsenosides Rg1 and Rg3, glycyrrhizin, isoliquiritigenin, vitamin E, vitamin C, and α-lipoic acid. In PD mitochondrially targeted antioxidants are one of the most effective therapies. Mitoquinone, melatonine, Sezto-Schiller tetrapeptides are the main compounds with pharmacological activity used for this pathology. Regarding ALS, the therapeutic strategy has been directed on the mitochondria to significantly reduce ROS production and inhibit the apoptotic pathways. To achieve this, drugs such as riluzole, creatine, coenzyme Q10, olesoxime, dexpramipexole, and minocycline are used. Concerning HD, antioxidants have been the most efficient bioactive compounds in both natural and synthetic therapeutics, such as withaferin A, withanolide A and withanolide D-P, bacosides A and B, asiaticoside, asiatic acid, madecassoside, naringin, hesperidine, kaempferol, perampanel, carotenoid, sesamol, vitamin C, α-lipoic acid, methazolamide, minocycline, tauroursodeoxycholic acid, and olanzapine. All these drugs are aimed at maintaining mitochondrial integrity, since this plays an important role in brain development and differentiation, plasticity, and neuronal activity, with direct or indirect pathologies mentioned above (AD, PD, ALS, and HD). Therefore, the administration of these therapeutic agents will depend on the mitochondrial dysfunction, the specificity of the pathology developed, and the patient’s idiosyncrasy.

## 6. Conclusions

The synthesis of ATP, carried out by the mitochondria, is essential for cellular homeostasis, so a change in mitochondrial activity modifies intracellular conditions. These modifications can range from changes in the balance of various metabolites to generating aging and early cell death. Ca^2+^ is one of the main second messengers and it participates in regulating different intracellular and intramitochondrial metabolic pathways. Its homeostasis is essential for cells to maintain the balance of various metabolic pathways, such as energy synthesis itself or mitochondrial dynamics. An imbalance or failure in energy generation results in the uncontrolled production of ROS, which leads to oxidative stress at the mitochondrial level. This stress condition leads to an overload of calcium or ROS that triggers the opening of the mPTP, leading to loss of mitochondrial membrane potential, decreased ATP production, and the release of mitochondrial proteins such as cytochrome c, which causes cell death through necrotic and apoptotic pathways.

Therefore, most studies on mitochondrial function are focused on discerning the interactions between ROS and calcium, since ROS can modify cellular calcium signaling. Furthermore, calcium signaling is essential for ROS production. Therefore, advances related to the mechanisms that regulate mitochondrial calcium levels have resulted in various therapies that can reduce mitochondrial dysfunction and thus prevent neurodegenerative diseases progression such as, Alzheimer’s, Parkinson’s, or Huntington’s diseases. However, much remains to be understood about the complicated mitochondrial calcium homeostasis and the molecular targets involved.

## Figures and Tables

**Table 1:** Some drugs targeting mitochondrial dysfunction.

Drug	Possible Mechanism of Action	Therapeutic Uses	References
**Olesoxime**	Scavenges the toxic by-products of lipid peroxidation. It reduced the overactivation of calpains.	Antioxidant and neuroprotective activities in NP, ALS, HD, PD, peripheral neuropathy, and spinal muscular atrophy.	[195]
**Cholest-4-en-3-one**	This drug is bound directly to two components of the mitochondrial permeability transition pore: the voltage-dependent anion channel and peripheral benzodiazepine receptor, suggesting a potential mechanism for its neuroprotective activity.	Effective in treating painful diabetic, chemotherapy-induced neuropathies, and ALS.	[195]
**Vitamin C**	Maintains the integrity of cell membranes and organelles, including mitochondrial membranes. This compound acts as a natural antioxidant.	Antioxidant and neuroprotective activities in NP	[197]
**N-acetyl-cysteine**	Reduce both excitotoxicity and oxidative stress through its actions on glutamate reuptake and antioxidant capacity.	It showed antioxidative properties measured by increased blood and brain glutathione levels after single-time point administration	[198,199]
**Apocynin**	Prevents mitochondrial dysfunction as an NADPH oxidase (NOX) inhibitor. Recent studies highlight its off-target effects. It can function as a scavenger of non-radical oxidant species, which is relevant for its activity against NOX 4 mediated hydrogen peroxide production.	Has been adjusted to target mitochondria (Mito-Apo), with preclinical PD models showing that it could prevent mPTP-induced nigral cell loss, indicating its potential use for mitochondrial dysfunction in PD. Reduce ROS successfully and uses various disorders, such as diabetic complications, neurodegeneration, cardiovascular disorders, lung cancer, hepatocellular cancer, pancreatic cancer, and pheochromocytoma.	[200,201]
**N-Methyl, N-propynyl-2-phenylethylamine (MPPE)**	Maintains the integrity of cell membranes and organelles, including mitochondrial membranes. MPPE is a propargylamine-based monoamine oxidase B (MAO-B) inhibitor.	MPPE serves as an MAO-B inhibitor that prevents MPTP-induced nigral cell loss, upregulates mitochondrial superoxide dismutase to alleviate oxidative stress, and improves complex I (CI) function	[202]
**S(-) enantiomer of pramipexole**	Balance the redox state levels of the cell and is a dopamine agonist.	Using dopamine agonists in routine clinical care has also had antioxidative properties. Used in the symptomatic treatment of PD.	[203]
**Ursodeoxycholic acid**	Shown to prevent mitochondrial membrane depolarization and stabilizes cytochrome c in the mitochondrial membrane.	A drug often used in chronic inflammatory liver disease with an extensive safety profile.	[204,205]
**Vitamin E**	Maintains the integrity of cell membranes and organelles, including mitochondrial membranes, by inhibiting peroxidation of the lipids that make up said membranes.	Antioxidant properties in AD and PD.	[206,207]
**α-Lipoic acid**	Acts as a potent antioxidant and decreased a marker of oxidative stress.	Antioxidant properties in AD.	[206,207]
**Olanzapine**	It has affinity for dopamine D1 and D2 receptors, as well as serotonin 5-HT2A receptors.	Antipsychotic agent for the treatment of schizophrenia and other disorders.	[208]
**Mitoquinone**	Produces direct antioxidant action by scavenging peroxyl, peroxynitrite and superoxide. Is a mitochondria-targeted antioxidant that reduces mitochondrial overproduction of ROS.	Antioxidant properties in PD and NDDs.	[209]
**Melatonin**	Direct scavenger of many ROS species such as free radicals, peroxylnitrites, hydroxyls, peroxyls, and other nitrous oxides under normal conditions.	It is mainly used as a dietary supplement for sleep regulation and re-synchronization of disrupted circadian rhythms and antioxidant properties in PD.	[210]
**Riluzole**	Reduces ROS generation via induction of glutathione production. Inhibits the release of glutamic acid from cultured neurons, from brain slices, and from corticostriatal neurons in vivo.	Antioxidant properties in ALS and as neuroprotective, anticonvulsant, and sedative properties.	[179]
**Dichloroacetate**	Dichloroacetate activates the pyruvate dehydrogenase complex and lowers cerebral lactate amounts.	Neuroprotective activity in HD and treatment of mitochondrial genetic diseases.	[208]
**Z-FA-MK**	Anti-apoptotic in function, it also inhibits effector caspases.	Effective therapeutic target in MS.	[211]
